# Ultra-broadband metamaterial absorbers from long to very long infrared regime

**DOI:** 10.1038/s41377-021-00577-8

**Published:** 2021-07-05

**Authors:** Yu Zhou, Zheng Qin, Zhongzhu Liang, Dejia Meng, Haiyang Xu, David R. Smith, Yichun Liu

**Affiliations:** 1 State Key Laboratory of Applied Optics, Changchun Institute of Optics, Fine Mechanics and Physics, Chinese Academy of Sciences, 130033 Changchun, Jilin China; 2 University of Chinese Academy of Sciences, 100049 Beijing, China; 3 Center for Advanced Optoelectronic Functional Materials Research and Key Laboratory of UV Light-Emitting Materials and Technology of Ministry of Education, College of Physics, Northeast Normal University, 130024 Changchun, China; 4 Center for Metamaterials and Integrated Plasmonics, Duke University, P.O. Box 90291, Durham, NC 27708 USA

**Keywords:** Optics and photonics, Metamaterials

## Abstract

Broadband metamaterials absorbers with high absorption, ultrathin thickness and easy configurations are in great demand for many potential applications. In this paper, we first analyse the coupling resonances in a Ti/Ge/Ti three-layer absorber, which can realise broadband absorption from 8 to 12 μm. Then we experimentally demonstrate two types of absorbers based on the Ti/Ge/Si_3_N_4_/Ti configuration. By taking advantage of coupling surface plasmon resonances and intrinsic absorption of lossy material Si_3_N_4_, the average absorptions of two types of absorbers achieve almost 95% from 8 to 14 μm (experiment result: 78% from 6.5 to 13.5 μm). In order to expand the absorption bandwidth, we further propose two Ti/Si/SiO_2_/Ti absorbers which can absorb 92% and 87% of ultra-broadband light in the 14–30 μm and 8–30 μm spectral range, respectively. Our findings establish general and systematic strategies for guiding the design of metamaterial absorbers with excellent broadband absorption and pave the way for enhancing the optical performance in applications of infrared thermal emitters, imaging and photodetectors.

## Abstract (web summary)

Ultra-broadband metamaterials absorbers with high absorption, ultrathin thickness and easy configurations are designed and demonstrated, which pave the way for enhancing the optical performance in applications of infrared thermal emitters, imaging and photodetectors.

## Graphical abstract

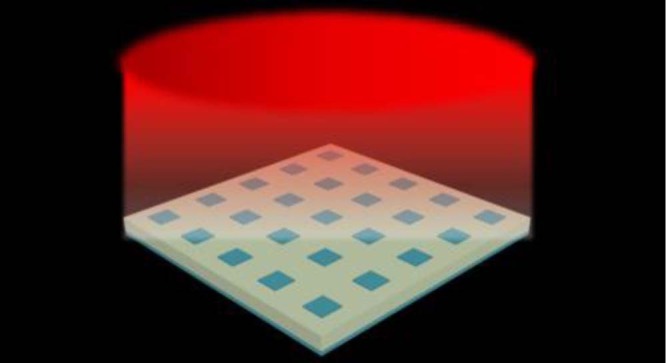

## Introduction

Metamaterials are made from artificially structured composite materials with numerous exotic properties which cannot be obtained from naturally occurring materials. They can yield a desired permittivity or permeability through adjustment of the size and geometry of structures which have a variety of applications in the field of light trapping and manipulation. Metamaterials’ electromagnetic properties are described based on the effective parameters. This characteristic gives metamaterials great potential in a variety of applications including light manipulation^1–3^, imaging^4–6^, sensing^7–9^, super-lenses^10,11^ and several other processes, among which field–metamaterial perfect absorbers (MPAs) have been a topic of considerable research interest in recent years. MPAs generally consist of metal and dielectric elements with dynamic control features of only subwavelength thicknesses. Initial MPAs normally consist of a three-layer structure that is known as a metal/insulator/metal (MIM) configuration. Special absorbers with fixed absorption performance have great potential for a variety of important scientific and technical applications, including thermal emitters^12,13^, energy harvesting^14–16^ and detection^17,18^.

Due to light-manipulation’s characteristics, MPAs only work at limited frequencies or wavelengths by adjusting their resonance features. Since Landy et al. demonstrated the first metamaterial absorber with one perfect absorption peak in the microwave band through an experiment^19^, the control and engineering of the spectral absorption properties of MPAs has attracted significant interest. In their experiment, broadband perfect absorption was achieved with MPAs in microwave regime^20–24^ and was extended from visible^25–37^, infrared^38–51^ and THz^52–57^ bands. This study will compare these representative theoretical and experimental works on the topic of broadband MPAs from visible to infrared bands in supplement document. Research on broadband absorbers has led to the creation of a number of methods which are successfully used for expanding the absorption band using laminated structures^33,36,41^, coupling multi-sized metallic resonators ^40,42,45,50^ and inlaying metallic structures in the dielectric layer^25,27,29,31,49^. The common absorption mechanism of these traditional methods is coupling multiple resonances of broad wavelength bandwidth excited by multi-sized metallic resonators in the structures. However, these methods for the achievement of broad absorption suffer due to the need for large and complicated structures, as they require large unit cells for containing the multi-sized resonators which are necessary for ensuring sufficient resonance to enhance absorption in the target regimes. With previous studies on broadband metamaterial absorbers, a significant majority of reports on broadband absorbers has focused on visible, short-infrared and THz regimes. There are few reports on ultra-broadband absorbers from the long to very long regime (8–30 μm). Studies of 8–12 or 8–14 μm relate to long-wavelength infrared (LWIR) imaging and detection, and with the very long infrared regime (14–30 μm), the applications are interstellar target detection in space and tracking detection for missiles, satellites and other aircraft. Another kind of broadband absorbers is silicon-based materials with specific surface morphology fabricated by laser ablation^58^ or plasma reactive ion etching^59^. Such metamaterials possess high scalability in imaging and photodetection due to the silicon-based substrate, as well as relatively low cost due to the mask-free and pattern-free processing. However, the dielectric thicknesses of metamaterial absorbers with MIM configurations can be designed thinner. For some applications such as integrating absorbers onto uncooled infrared detectors and microbolometers, an ultrathin broadband absorber is required. An absorber with a thick dielectric can obtain good absorptivity but increases thermal capacity, and greater thermal capacity implies more noise equivalent temperature difference (NETD)^60,61^. Due to the wide wavelength bandwidth, the absorbers’ unit cells are usually large with complicated structures. So the designing of absorbers with high absorption, ultrathin thickness and easy configurations is quite a breakthrough.

In this paper, a new design strategy to achieve ultra-broadband absorbers targeting extended wavelength band from long to very long-wavelength infrared regimes (8–30 μm) will be demonstrated. For applications including the integration of absorbers on to uncooled infrared detectors and microbolometers, small and easy absorber structures can reduce fabrication costs and noise equivalent temperature difference (NETD). By focusing on realising perfect broadband absorption from the long to very long infrared band and revealing the designing theories, the excitation conditions of propagating and localised surface plasmon resonances in a Ti/Ge/Ti three-layer absorber will be analysed, which can achieve average absorption of 90% (experiment result: 80%) in the range of 8–12 μm. An experiment will then be conducted demonstrating two types of four-layer absorbers based on the Ti/Ge/Si_3_N_4_/Ti configuration. The proposed Ti/Ge/Si_3_N_4_/Ti absorber has a dielectric section consisting of laminated dielectric layers made from lossless and lossy materials, intending the excitation of multiple absorption mechanisms. The average absorptions of two types of Ti/Ge/Si_3_N_4_/Ti absorbers achieve to be nearly 95% from 8 to 14 μm (experiment result: 78% from 6.5 to 13.5 μm). The metal layers are constructed from intrinsically lossy metals. With similar metasurface design and absorption mechanisms, Ti/Si/SiO_2_/Ti absorbers which absorb 92% and 87% of ultra-broadband light in the 14–30 μm and 8–30 μm spectral range are proposed. For the dielectric layers of both structures, a combination of a lossless material (either Ge or Si) on top and a lossy material (either Si_3_N_4_ or SiO_2_) on bottom are chosen in order to achieve the best coupling of PSPR- and LSPR-dominated absorption. In addition, the intrinsic absorption of lossy dielectric Si_3_N_4_ or SiO_2_ layer also expands the absorption bandwidth. Due to unique metasurface structures and absorption mechanisms, the absorbers in this experiment can achieve prominent absorption abilities in target infrared wavelength regimes, with small and easy configurations capable of reducing fabrication costs for certain potential applications, including thermal emitters, infrared imaging and photodetectors.

## Results

### Coupling plasmon resonances in the Ti/Ge/Ti absorber

The entire absorber with a MIM configuration can be seen as a resonant cavity enclosed in magnetic walls which support a variety of resonant modes. When the top metallic layer is hit with incident light, the bottom metal layer acts as a mirror which reflects the surface plasmon excited at the lower metal-insulator interface. The destructive interference of the surface plasmon between the upper and lower metal-insulator interfaces produces resonant modes in the dielectric cavity, thereby causing light to become trapped and absorbed. Maximisation of the absorption of the target band while minimising unwanted reflections is possible through adjustment of the MIM absorber’s geometric parameters which ensures sufficient energy coupling in various resonant modes. The bottom metallic layer’s thickness is considerably greater than the skin depth, which prevents transmission. The insulator layer’s thickness is far less than the radiant wavelength, which enables effective coupling of the surface plasmons in the dielectric cavity. However, if the dielectric thickness is too little, a significant amount of reflection will occur. As is widely known, when the effective impedance of the absorber matches free space, reflection reaches a minimum. At the same time, if the thickness of the bottom metal ground plane is greater than the target incident wave’s penetration depth, almost zero transmission will be caused. Perfect absorption can then be achieved. Absorption is expressed as^19^:

1 $$A\left( \lambda \right) = 1 - R\left( \lambda \right) = 1 - \left| {\frac{{Z - Z_0}}{{Z + Z_0}}} \right|^2 = 1 - \left| {\frac{{\sqrt \mu - \sqrt \varepsilon }}{{\sqrt \mu + \sqrt \varepsilon }}} \right|$$

where *Z*_0_ and *Z* are the effective impedance of free space and the absorber, and *μ* and *ε* are the effective permeability and permittivity of the absorber. This shows that *ε* = *μ* is a critical factor for the achievement of perfect absorption. In addition, to match impedance to free space, electric and magnetic resonances are of equal importance. Metals are requisite components of the MPAs of MIM designs, consisting of the top periodic structures and the bottom layers. In the LWIR band, MPA’s top metallic layer does not behave similarly to the DC surface resistances, so therefore, the Drude model can be used for calculating the resonant characteristics, and the metallic layer’s permittivity is expressed as^62^:

2 $$\varepsilon _m = 1 - \frac{{\omega _p^2}}{{\omega ^2 + i\gamma \omega }}\left\{ {\varepsilon _1 = \frac{{\omega _p^2}}{{\omega ^2 + \gamma ^2}},\,\varepsilon _2 = \frac{{\omega _p^2\gamma }}{{\omega \left( {\omega ^2 + \gamma ^2} \right)}}} \right\}$$

where *ε*_1_ and *ε*_2_ are the real and imaginary parts of permittivity, *ω*_*p*_ is the plasmon frequency and *γ* is a damping constant.

**Fig. 1:**
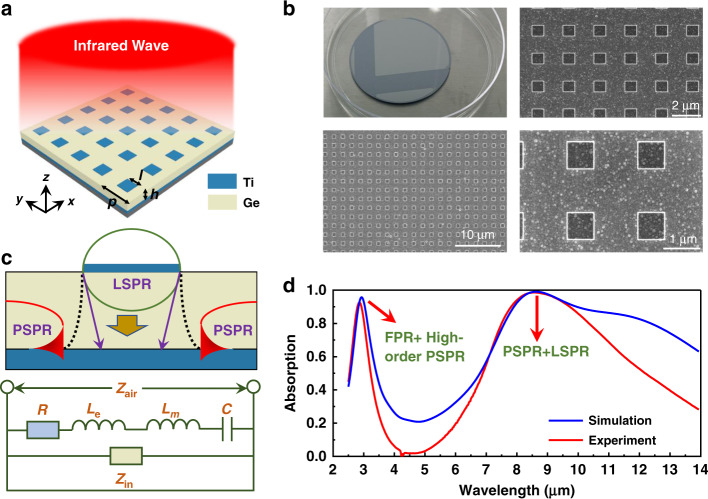
Absorption characteristics of the Ti/Ge/Ti absorber. **a** Schematic of the Ti/Ge/Ti absorber with periodic square top resonators. In this design, *l* = 0.8 μm, *p* = 2 μm and *h* = 0.5 μm. The thickness of the top Ti layer is 22 nm, and the bottom Ti layer is 120 nm, where is thicker than its penetration depth. **b** SEM images of the fabricated Ti/Ge/Ti absorber. **c** Schematic of different resonant modes and equivalent RLC circuit of the Ti/Ge/Ti unit cell. **d** Simulated and experiment absorption spectra of the Ti/Ge/Ti absorber

In recent years, numerous new methods have been attempted to achieve broadband absorption (see Table S1 in Supplement document). Initially, the designs of the metallic layers of MPAs were limited mostly to high-Q-factor noble metals, including Au^25,28,38,39,51^ and Ag^27,40^, which caused excellent resonant selectivity. Recently, it has been discovered that some refractory metals can excite broadband plasmon resonances, including Mo^43^, W ^33,35,37,45^ and Ti^30,34,35,48–50^. In the band from visible to mid-infrared wavelengths, these metals have a large imaginary-part in their refractive indices, and have highly lossy properties which contribute to broadband absorption. Some metal compounds, including TiN, can also be used for the top layers of MPAs^26,31^. In this section, the aim is to reveal the origins of broadband absorption in a Ti/Ge/Ti three-layer absorber, as can be seen in Fig. 1a, and a sample is fabricated which is based on our design (see Fig. 1b for images of the sample). Figure 1c shows the schematic of different resonant modes and equivalent RLC circuit of the Ti/Ge/Ti unit cell. For an MIM metamaterial absorber, two resonant modes commonly appear to affect the absorption characteristics, propagating surface plasmon (PSP) and localised surface plasmon (LSP) resonances. The absorption spectrum of the proposed Ti/Ge/Ti absorber is shown in Fig. 1d. With the simulation results, three absorption peaks can be seen in the mid-infrared (MIR) and long-wavelength infrared (LWIR) bands, located at 2.93 μm, 8.64 μm and 12.06 μm, and with respective aborptivities of 95.7%, 99.1% and 82.2%. With the experiment result, two absorption peaks can be seen at 2.86 μm and 8.53 μm, with absorptivities of 92.5% and 98.6%. Three absorption spectra of different sizes (0.6 μm, 0.8 μm and 1.0 μm lengths) of the top Ti nano-square resonators were measured (see Fig. S1a, b in supporting information). Compared to other metals, Ti has great potential and exhibits better performance when used as the metallic layer of infrared broadband MPAs^30,48–50^ (see Fig. S1d in supporting information). Although the simulated third peak is not appeared in actual measurement, it verifies that the absorption of the Ti/Ge/Ti absorber is dominated by coupling resonances as the experiment absorption spectrum is not symmetrical in the LWIR band. Generally, the proposed Ti/Ge/Ti absorber realises good responses in both MIR and LWIR bands.

Returning to Fig. 1c, there are two main resonances which enhance the broadband absorption. For propagating surface plasmon resonance (PSPR), a specially nonlocalised mode is produced by metal surface electrons when excited by incident light. According to Bloch’s theorem, in periodic structure, the wave vector of the wave function of quantum state plus one or several inverted lattice vectors has no effect on the wave function’s energy. Therefore, when the incident wave shines on the metal grating, one or several inverted grid vectors can be added to the wave vector of the incident wave. As a result, the wave vector following the addition of the inverted lattice vector satisfies the wave vector at the interface of the metal medium, thereby exciting the PSPs. For a three-layer MPA of MIM design, the periodic metallic structures of the top layer are regarded as a grating to match the wave vector of diffraction to the wave vector of PSPs, and PSPs are evanescent waves which propagate along the interface between the bottom metallic and the middle dielectric layer. The PSPR can be determined using the following equation^7^:

3 $$k_0n_d\sin \theta + 2\pi m/p - n_dh\cos \theta /m = k_0[\varepsilon _m(\lambda )n^{2/d}/(\varepsilon _m(\lambda )+ n_d^2)]^{1/2} = k_{PSP}$$

where *p* is the period of the absorber, *h* is the dielectric thickness, *ε*_*m*_ (*λ*) = *ε*_1_ + *iε*_2_ is the complex permittivity of the bottom metallic layer, *n*_*d*_ is the refractive index of the dielectric layer, *θ* is the incident angle and *m* is an integer which represents the order of the PSPR. Localised surface plasmons (LSPs) are localised modes which exist near the insulator’s top metallic structure and gradually diffuse into the entire insulator. Localised surface plasmon resonance (LSPR) can be numerically expressed and illustrated using an *RLC* circuit mode. The transmission line model and simplified circuit model of a single resonator of a unit cell are shown in Fig. 1c. It is also assumed that the adjacent unit cell nanostructures in the metamaterial are far enough apart for the prevention of electromagnetic coupling. From transmission line theory, it is assumed that the subwavelength component is an equivalent circuit with multiple *RLC* lumped elements. In this circuit, the lumped elements include the ohmic resistance: *R*; the kinetic inductance: *L*_e_, which is induced by the drifting electrons generally found within the skin depth of the metallic layer; the Faraday inductance: *L*_m_, which is due to the mutual effects between the two metallic layers; and the equivalent capacitance: *C*, for the insulator between the two metallic layers. These are represented as^40^:

4 $$R = \frac{{\gamma l}}{{\varepsilon _0pt\omega _{p{\mathrm{1}}}^2}} + \frac{\gamma }{{\varepsilon _0\delta _b\omega _{p{\mathrm{2}}}^2}}$$

5 $$L_{\mathrm{m}} = \mu _0hl/p$$

6 $$L_{\mathrm{e}} = \frac{l}{{\varepsilon _0pt\omega _{p1}^2}} + \frac{1}{{\varepsilon _0\delta _b\omega _{p2}^2}} = \frac{1}{{\varepsilon _0\omega ^{\mathrm{2}}}}\left[ {\frac{{l\varepsilon _{1t}}}{{pt\left( {\varepsilon _{1t}^2 + \varepsilon _{2t}^2} \right)}} + \frac{{\varepsilon _{1b}}}{{\delta _b\left( {\varepsilon _{1b}^2 + \varepsilon _{2b}^2} \right)}}} \right]$$

7 $$C = \alpha \varepsilon _r\varepsilon _0pl/h$$

where *ε*_0_ and *μ*_0_ denote the permittivity and permeability of a vacuum; *ε*_r_ represents the relative permittivity of the insulator; *ω*_*p*1_, *ω*_*p*2_, *ε*_1*t*_, *ε*_2*t*_, *ε*_1*b*_ and *ε*_2*b*_ are the plasmon frequency, the real and imaginary parts of the permittivity of the top and bottom metallic layers; *α* is the tuning factor of the equivalent capacitance for the gap; *h* is the dielectric thickness; *p* is the period of the MPA; *l* is the effective resonant length of the top metallic layer, *t* is the thickness of the top metallic layer and *δ*_*b*_ is the penetration depth of the bottom metallic layer. The thickness of the top metallic layer is less than the penetration depth. The impedance of the *RLC* model is expressed as:

8 $$Z_{LC} = R + \frac{{2\varepsilon _i}}{{\omega C\left( {\varepsilon _{\mathrm{r}} + 1} \right)^2}} + i\omega \left( {L_{\mathrm{m}} + L_{\mathrm{e}}} \right) + \frac{2}{{i\omega C\left( {\varepsilon _{\mathrm{r}} + 1} \right)}}$$

where *ε*_r_ and *ε*_i_ are the real and imaginary parts of the insulator’s relative permittivity. The first term represents the metallic loss caused by joule heating of the top metallic structures, the second term represents the dielectric loss of the insulator layer and the third and fourth terms are the reactance from the equivalent inductance and capacitance of the top metallic structures. The insulator layer is similar to a transmission line with a length that is equal to the thickness of the spacer and shorted by the ground plane, where its impedance is represented as:

9 $$Z_{in} = i\sqrt {\frac{{\mu _d}}{{\varepsilon _d}}} Z_0\tan \left( {k_0\sqrt {\varepsilon _d\mu _d} h} \right)$$

where *Z*_in_ is the impedance of the shorted transmission line, *k*_0_ is the free space wave number, *μ*_d_ and *ε*_d _ = *ε*_r_ + *iε*_i_ are the relative permeability and permittivity of the insulator and *h* is the thickness of the insulator layer. Based on the equivalent circuit model which can be seen in Fig. 1c, the metamaterial absorber’s overall impedance is expressed as:

10 $$Z_{MPA} = \frac{{Z_{LC}Z_{in}}}{{Z_{LC} + Z_{in}}}$$

When the overall impedance of the absorber perfectly matches that of the free space, the reflection is reduced to a minimum and the achievement of perfect absorption is possible. therefore, the LSPR wavelength is obtained as below:

11 $$\lambda _{LSP} = 2\pi c\sqrt {\frac{{\left( {L_e + L_m} \right)C}}{m}} {\mathrm{ + }}\frac{{n_dh\cos \theta }}{m}$$

where *m* is an integer that represents the order of the LSPR, which implies that high-order LSP modes can be excited in the target band and that their resonant wavelengths are smaller than those of the fundamental LSP mode. Generally, when designing a metamaterial absorber that operates in the LWIR band, a sharp absorption spectrum is produced in mid-infrared region, which is the high-order LSPR-dominated absorption peak. In addition, the second term of Eq. (11) represents the phase difference which is induced by the absorber’s dielectric layer, thereby showing that the LSP resonant wavelengths are also impacted by the dielectric thickness.

**Fig. 2:**
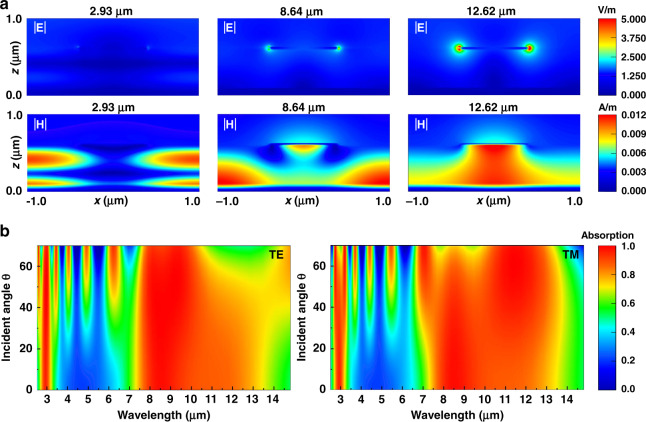
Analyses of electromagnetic field distributions and absorption spectra for different incident angles. **a** Electromagnetic field distributions at the three resonant wavelengths along the MIR and LWIR range: 2.93 μm, 8.64 μm and 12.62 μm. **b** TE-mode (x-polarised) and TM-mode (y-polarised) absorption spectra for incident angles from 0 to 70°

To show the coupling resonances of the Ti/Ge/Ti absorber, the electromagnetic field distributions were simulated at the three resonant wavelengths (2.93 μm, 8.64 μm and 12.62 μm) of the simulated absorption spectrum along the MIR and LWIR range. As Fig. 2a shows, the electric field is strongly confined to the air-slot of the upper Ti structure, which implies that the surface plasmon polaritons (SPPs) are excited in the metamaterial absorber. Two different resonant modes can be seen in the magnetic field distributions. At a wavelength of 2.93 μm, Fabry-Pérot mode and high-order PSP resonance coexist in the dielectric layer which enhance absorptivity (from Fig. S1c, d, Fabry-Pérot resonance is sensitive to dielectric thickness and not very sensitive to period of the absorber. Changing the bottom layer with different metals can separate the absorption peaks dominated by Fabry-Pérot and high-order PSP resonances). At a wavelength of 8.64 μm, the resonance is considered as the fundamental PSPR between the continuous Ti film and Ge spacer, and the magnetic field is not only strongly confined to the gap region underneath the nano-cubes but is also intensively enhanced between the adjacent cells. As the wavelength increases, the PSPR gradually disappears. At a wavelength of 12.62 μm, the LSPR excited by the periodic Ti nano-cube structures dominate absorption. The spectra of absorption with oblique incidences for TE-polarised (*φ* = 0°) and TM-polarised (*φ* = 90°) waves can be seen in Fig. 2b. At small incident angles, it can be observed that the proposed absorber exhibits almost angle-independent absorption for both the TE- and TM-modes, and the absorption spectrum remains almost completely unchanged for both polarisations with incident angles up to 20°. For large incident angles, the absorber behaves differently for the TE- and TM-polarised waves. As the incident angle increases, the average absorptivity and bandwidth gradually decrease with fluctuations. The incident magnetic field’s horizontal component decreases as the incident angle increases, and the strength of the coupling and absorption weakens. Figure 2b shows that the proposed Ti/Ge/Ti absorber can excite high-order PSP and LSP modes at large incident angles in the region from the MIR to the LWIR band.

### Ti/Ge/Si_3_N_4_/Ti near-perfect broadband LWIR absorbers

**Fig. 3:**
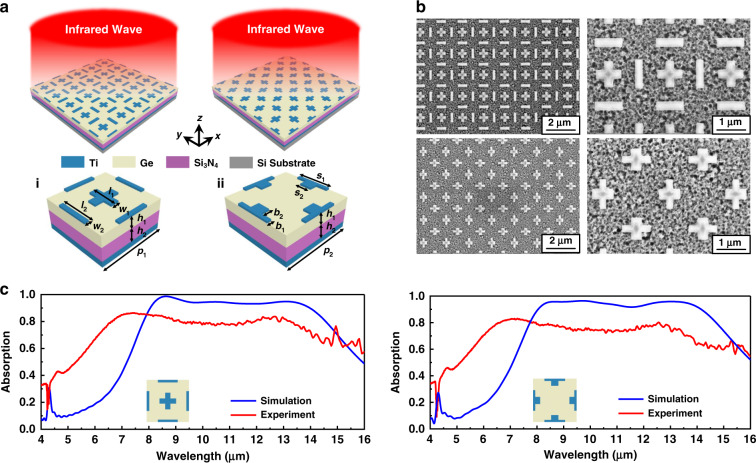
Absorption characteristics of two types of Ti/Ge/Si_3_N_4_/Ti absorbers. **a** Schematic of two types of Ti/Ge/Si_3_N_4_/Ti absorbers (*h*_1_ = 270 nm, *h*_2_ = 330 nm, *p*_1_ = 1.6 μm and *p*_2_ = 1.8 μm): (i) the top layer consists of periodic nano-cross and nano-strip structures (*l*_1_ = 630 nm, *w*_1_ = 200 nm, *l*_2_ = 750 nm and *w*_2_ = 100 nm); (ii) the top layer consists of irregular nano-cross structures (*s*_1_ = 850 nm, *b*_1_ = 100 nm, *s*_2_ = 300 nm and *b*_2_ = 200 nm). The thickness of the top Ti layer is 22 nm, and the bottom Ti layer is 120 nm, where is thicker than its penetration depth. **b** SEM images of two types of fabricated Ti/Ge/Si_3_N_4_/Ti absorbers. **c** Simulated and experiment absorption spectra of the two types of Ti/Ge/Si_3_N_4_/Ti absorbers

In this section, a new design for the realisation of near-perfect broadband absorption in the entire LWIR band is outlined. This method adjusts the components of the dielectric layers of MPAs. Here, a method is proposed which constructs the dielectric layer from two plane layers of lossless and lossy materials. The lossless dielectric layer possesses a constantly high real part of the refractive index and a zero imaginary part, whereas the lossy dielectric layer has an imaginary part of the refractive index in the target band which is capable of absorbing a portion of the incident wave. Compared to other reported absorbers with lossy materials, including SiO_2_^48^ and Si_3_N_4_^27,31^, the designs used here consist of composite dielectric layers of lossless and lossy materials, which is able to realise and combine intrinsic absorption and hybrid plasmon resonances in order to achieve more perfect broadband absorption in the target LWIR regime. Based on the initial MIM configuration, two types of Ti/Ge/Si_3_N_4_/Ti four-layer absorbers are proposed, which are shown in Fig. 3a, consisting of a top layer of periodic Ti resonators array, two dielectric layers of Ge and Si_3_N_4_ and a bottom layer of Ti. The corresponding SEM images and absorption spectra are displayed in Fig. 3b. Simulated and experiment absorption spectra of the two types of Ti/Ge/Si_3_N_4_/Ti absorbers are shown in Fig. 3c. From the simulation results, the two proposed types of Ti/Ge/Si_3_N_4_/Ti absorbers exhibited high absorptions of over 90% in a broad wavelength spread from 8 to 14 μm, covering the whole LWIR regime (spectra of absorption with oblique incidences for TE-and TM-polarised waves can be seen in Fig. S2). Absorption characteristics of different thickness of two dielectric layers are compared in Fig. S3. For such absorbers with lossy dielectric layers, the imaginary-parts refractive index of Si_3_N_4_ govern the excitation of the intrinsic absorption of incident light at LWIR regime, and this part of absorption can be coupled with plasmon resonances and jointly enhances broadband absorption. For broadband absorbers with ultrathin dielectric thicknesses, it needs the designers to choose high refractive index materials to control the resonant bands to locate at target wavelength regimes. Ge have large refractive index in the infrared regime, which only requires thin thickness control PSPR- and LSPR-dominated absorption to take place in the large wavelength band. In other words, the Ge layer keeps high refractive index for the total dielectric. The experiment average absorptions for the two absorbers are nearly 78% for the wavelengths of 6.5–13.5 μm. For the gaps between simulation and experiment results of the Ti/Ge/Si_3_N_4_/Ti absorbers, Firstly, according to previous experiment absorption results involving the Ti/Ge/Ti absorber, the actual refractive index of fabricated Si_3_N_4_ can cause a part of deviations for the resonant wavelengths; Secondly, the diaphragm of the measuring system is chosen as 50 × 50 μm, there are some measurement errors for the reflected power of the Au background and the structures at such small size of light source; Lastly, the sizes of actual fabrications may appear some errors such as inaccurately control of film thicknesses. In general, compared to three-layer MIM absorbers, the Ti/Ge/Si_3_N_4_/Ti with adjustable parameters can expand the absorption band in target LWIR regime.

**Fig. 4:**
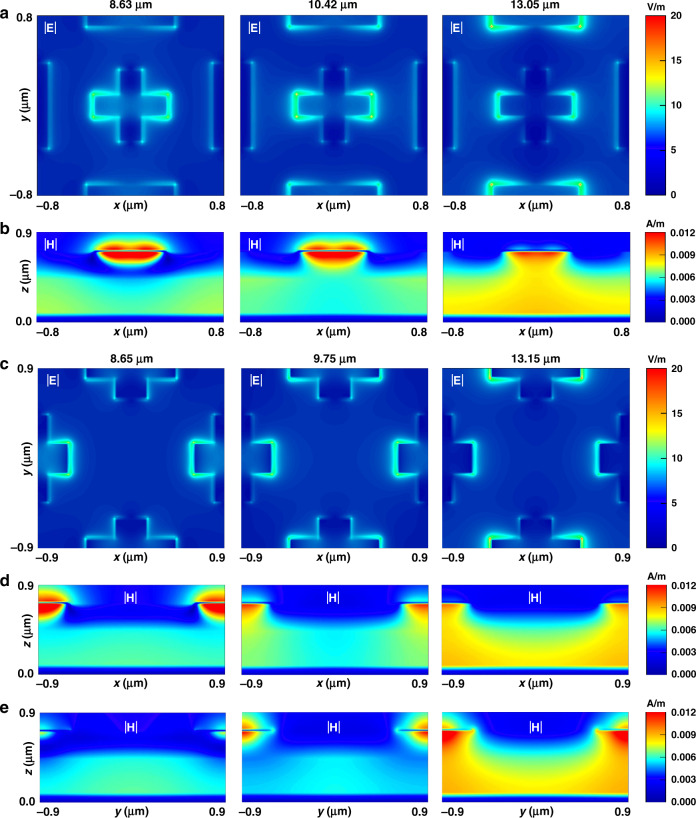
Electromagnetic field distributions of the two types of Ti/Ge/Si_3_N_4_/Ti absorbers at their three resonant wavelengths along the LWIR range. Ti/Ge/Si_3_N_4_/Ti absorber with periodic nano-cross and nano-strip top metallic resonators: **a** electric field distributions in the top layer (x-y plane). **b** Magnetic field distributions in the dielectric (x-z plane, *y* = 0). Ti/Ge/Si_3_N_4_/Ti absorber with periodic irregular nano-cross top metallic resonators: **c** electric field distributions in the top layer (x-y plane). **d** Magnetic field distributions in the dielectric (x-z plane, *y* = 0). **e** Magnetic field distributions in the dielectric (y-z plane, *x* = 0)

There are differences to the absorption mechanisms of both absorbers. Figure 4 displays both absorbers’ electromagnetic field distributions at their three absorption peaks. The incident wave is given a TM polarisation, which is a linear light that is parallel to the electric field incident in the x direction. According to the top layer’s electric field distribution in Fig. 4a, c, the top periodic metallic structures provide sustained resonances for the target LWIR band. When the incident waves of the target wavelength regime shoot on the absorber, the top metallic layers with uniquely adjustable and flexible cross resonator parameters are able to excite resonances which enhance broadband absorption in the target LWIR band. For the Ti/Ge/Si_3_N_4_/Ti absorber with periodic nano-cross and nano-strip top metallic resonators, the simulation results show three absorption peaks, located at 8.63 μm, 10.42 μm and 13.05 μm, with respective absorptions of 98.7%, 94.5% and 95.2%. Figure 4a shows that the periodic nano-cross and nano-strip top metallic structures provide sustained resonances for the target LWIR band. In Fig. 4b, at a wavelength of 8.63 μm, it can be seen that PSR resonances are found at the borders and in the lower part of the dielectric layer. As the incident wavelength increases, the LSP resonances excited by the nano-cross and nano-strip resonators gradually increase, thereby reducing the PSPR and dominating absorption. This indicates that the three absorption peaks are excited by PSP resonant mode and LSP resonant modes of nano-cross and nano-stripe resonators. For the Ti/Ge/Si_3_N_4_/Ti absorber with periodic irregular nano-cross top metallic resonators, the simulation results show three absorption peaks, located at 8.65 μm, 9.75 μm and 13.15 μm, with maximum respective absorptivity levels of 95.9%, 96.3% and 96.2%. Unlike with traditional symmetrical cross resonators that are used in the absorbers, each cross resonator in our structure is not symmetrical, but special arrangement of cross resonators consists of a symmetric unit cell leads to a polarisation–independent absorption characteristic. The lateral magnetic field distributions in the dielectric of the x-z and y-z planes at three resonant wavelengths can be seen in Fig. 4d, e. It can therefore be concluded that both absorption peaks at 8.65 μm and 9.75 μm are mainly dominated by x- and y-oriented PSP modes, and that the third absorption peak at 13.15 μm is dominated by LSP modes. Unlike the previous Ti/Ge/Si_3_N_4_/Ti absorber with periodic nano-cross and nano-strip top metallic resonators and other designs of ultra-broadband absorbers^30,49^ that utilise the absorption mechanism of coupling PSPR and LSPR with only one PSPR-dominated absorption peak, the unique design of top metallic cross structures is able to excite PSPs in order to propagate along x and y directions, producing two vertical directions of PSP resonances. Both directions of PSP resonance cooperatively dominate the absorptivity to realise broadband absorption before the induction of LSPs. Due to the flexible parameters of *s*_1_, *s*_2_, *b*_1_ and *b*_2_, the LSP modes of two y-direction cross resonators make up the total resonances for enhancing absorption.

### Ti/Si/SiO_2_/Ti absorbers from the long to very long infrared band

**Fig. 5:**
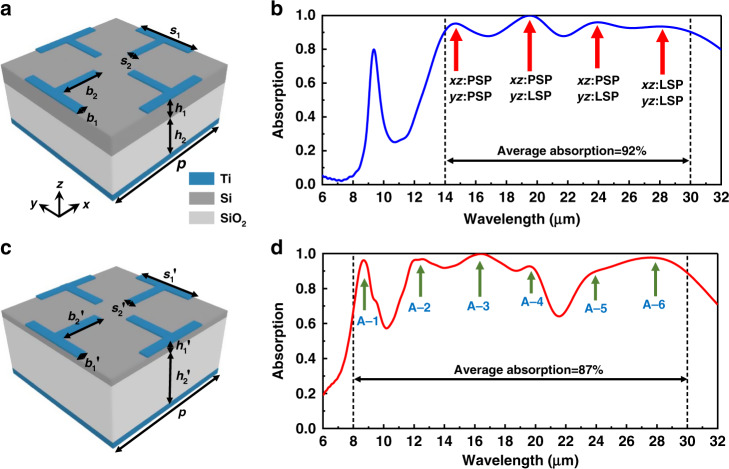
Simulated absorption characteristics of two types of Ti/Si/SiO_2_/Ti absorbers from long to very long infrared regime. **a** Schematic and **b** absorption spectrum of the Ti/Si/SiO_2_/Ti absorber from 14 to 30 μm. (*s*_1_ = 1.9 μm, *s*_2_ = 0.23 μm, *b*_1_ = 0.25 μm, *b*_2_ = 1.17 μm, *h*_1_ = 0.52 μm, *h*_2_ = 1.11 μm and *p* = 4 μm). **c** Schematic and **d** absorption spectrum of the Ti/Si/SiO_2_/Ti absorber from 8 to 30 μm. (*s*_1_′ = 1.9 μm, *s*_2_′ = 0.27 μm, *b*_1_′ = 0.25 μm, *b*_2_′ = 1.21 μm, *h*_1_′ = 0.18 μm and *h*_2_′ = 1.62 μm) The thickness of the top Ti layer is 22 nm and the bottom Ti layer is 120 nm, which is thicker than its penetration depth

Research on the very long-wavelength infrared (VLWIR) regime has some application potential for space exploration. However, very few accurate measuring devices exist for VLWR applications. Based on previous unique top resonators embedding a dual-dielectric layer stack, a new metasurface design is proposed, composed of a Ti/Si/SiO_2_/Ti configuration, as can be seen in Fig. 5a. A laminated dielectric is chosen with silicon (Si) and silicon dioxide (SiO_2_) planar layers. The absorption spectra are displayed in Fig. 5b, indicating that this type of structure has the ability to achieve near-perfect absorption by covering the whole VLWIR regime. The average absorption level is calculated as being 92% from 14 to 30 μm by using the simulation tool. Four peaks at 14.54 μm, 19.64 μm, 23.95 μm and 28.30 μm can be seen, with absorptivity of 95.23%, 99.92%, 95.20% and 93.76% (electromagnetic field distributions at their absorption peaks can be seen in Fig. S5). From absorption spectrum of the first Ti/Si/SiO_2_/Ti absorber, it can be seen that such ultra-broadband absorption covered the entire VLWIR regime. This indicates that periodic irregular nano-cross top resonators are theoretically capable of exciting four resonant modes (x-, y-direction PSP and LSP resonances). Through an adjustment of the structure’s parameters, as displayed in Fig. 5c, the Ti/Si/SiO_2_/Ti absorber can realise ultra-broadband absorption from 8 to 30 μm covering the entire LWIR and VLWIR regimes. The absorption spectrum is displayed in Fig. 5d. The average value of absorption from 8 to 30 μm is calculated as being 87%. Six absorption peaks of A–1, A–2, A–3, A–4, A–5 and A–6 can be seen sat wavelength of 8.68 μm, 12.57 μm, 16.44 μm, 19.59 μm, 23.53 μm and 27.59 μm with absorptivities of 96.18%, 96.78%, 99.78%, 92.72%, 87.80% and 97.69% (spectra of absorption with oblique incidences for TE- and TM-polarised waves can be seen in Fig. S7, and electromagnetic field distributions at their absorption peaks can be seen in Fig. S8). With the exception of the hybrid resonances from the x and y directions of PSP and LSP modes, the intrinsic absorption of SiO_2_ plays a vital role in the entire absorption bandwidth, mainly affecting and dominating the absorption peaks A–1 and A–4. By taking advantages of the PSP and LSP resonances, and intrinsic absorption of SiO_2_, under the total dielectric thickness of 1600 nm, the Ti/Si/SiO_2_/Ti absorber can excite six absorption peaks and achieve excellent absorption bandwidths in the target LWIR and VLWIR regimes. However, there are two dips at around 10 and 22 μm. The average absorption is hard to optimise to be perfect with several absorption peaks. It is difficult to increase absorption peaks under small dielectric thickness and easy top metallic layer. In our opinion, the way to improve the performance of the total absorption band is increasing the dielectric thickness and trying more complicated top metallic structures.

## Discussion

In this paper, several metamaterial absorbers are proposed and demonstrated for verifying the coupling of various resonances and intrinsic absorption in order to enhance ultra-broadband absorption using small and simple structures which can reduce the fabrication costs and noise equivalent temperature difference (NETD) in some potential applications. Firstly, we demonstrated a Ti/Ge/Ti three-layer absorber which was based on traditional MIM configuration. With the unique refractory metal of Ti, the Ti/Ge/Ti absorber showed good response in the LWIR band, achieving an average absorption of 90% (experiment result: 80%) in the range of 8–12 μm. We then elaborated on a four-layer design in order to achieve ultra-broadband metamaterial absorbers based on embedding dual dielectric layer stacks (one lossless and one lossy) in an MIM structure which also embedded arrayed metallic cross resonators with azimuthal symmetry. This idea was implemented with two designs: Ti/Ge/Si_3_N_4_/Ti and Ti/Si/SiO_2_/Ti metamaterial absorbers were proposed for the realisation of polarisation-independent, near-perfect and ultra-broadband absorption from the long to very long infrared regime (8–14 μm, 14–30 μm and 8–30 μm). The laminated dielectric layers made from lossless and lossy materials play a vital role in broadband absorption bandwidth. Firstly, the wavelength-dependent imaginary parts of the refractive index of the lossy materials (Si_3_N_4_ and SiO_2_) govern the excitation of the intrinsic absorption of incident light at target wavelength regimes. Secondly, the lossless materials (Ge and Si) have large permittivity in the infrared regime, which only requires thin thickness control LSPR-dominated absorption to take place in the large wavelength band within the upper locations of the dielectric. Finally, the lossy materials (Si_3_N_4_ and SiO_2_) have smaller permittivity than the chosen lossless dielectric layers, potentially facilitating easier excitation of PSP resonances at the lower locations of the dielectric and enhancing absorption. By designing and adjusting the metamaterial absorbers’ parameters, the broadband absorption bandwidths can be successfully coupled through intrinsic absorption from the lossy dielectric layers (PSPR- and LSPR-dominated absorptions are enhanced by special top metallic resonators). This work has great potential in a variety of applications, including thermal emitters, infrared imaging and photodetectors.

## Materials and methods

### Simulations

The numerical absorption spectra of the absorbers and the electromagnetic field distributions of the absorption peaks were theoretically simulated using the finite difference time domain (FDTD) method. A unit cell of the investigated structure was simulated using periodic boundary conditions along the x and y axes and perfectly matched layers along the propagation of electromagnetic waves (z axis). A mesh accuracy of 8 was chosen and the calculation time was set as 5000 fs with an automatic shutoff minimum of 1 × 10^−5^. The refractive indices of Ti, Ge, Si and SiO_2_ were derived from Palik’s handbook^62^ and the refractive index of Si_3_N_4_ was derived from Kischkat^63^. Plane waves were launched from the top of the metasurface and the electromagnetic field distributions were illuminated using a TM-polarised plane wave. For oblique incidence, the incident angles varied from 0° to 70° with a step of 2°.

### Fabrication

Two types of metamaterial absorber structure were fabricated for this work, Ti/Ge/Ti and Ti/Ge/Si_3_N_4_/Ti structures. The substrates were silicon with a crystal orientation of 100 (TIR < 3 μm, BOW < 10 μm, Ra < 5 nm). For substate-cleaning, ultrasonic treatment with NMP solution was performed for 5 min, followed by ultrasonic treatment with water for 5 min, before blow drying was performed. Si substrates were coated with 120 nm Ti layer using evaporation coating equipment. For different dielectric layers of the proposed absorbers, 500 nm Ge, 330 nm Si_3_N_4_ and 270 nm Ge layers were coated at the Ti layers. The coating vacuum degree was <5 × 10^−5^ Pa and the coating rate was 2 A/s. The substrate was then pre-treated by HDMs and coated with PMMA electron beam photoresist. E-beam lithography system was used for defining the patterns of the top Ti layers. For strip-cleaning purposes, the structures were completely immersed in NMP solution and heated to 120 °C. After 2 min, the Ti which required peeling off was obviously dropped in the solution. A new solution was then used, where the structures were immersed for a further 20 min, subjected to ultrasonic treatment for 5 min and then rinsed with clean water. Finally, the structure was chipped into small pieces (without destroying the graphics area).

### Optical characterisations

An infrared Fourier transformation spectroscope measuring system was used for demonstrating the reflection spectra of the Ti/Ge/Ti and Ti/Ge/Si_3_N_4_/Ti absorbers. The measured reflection was referenced to an Au mirror in order to determine absolute reflectivity, *R*. As the transmission channel is completely cancelled by the opaque metal mirror, absorption according to *A* = 1–*R*–*T* was determined directly to be *A* = 1–*R*. The measurement range was 600–7500 cm^−1^.

## Supplementary information

Additional information for proposed absorbers
