# Supplementary material for: Ultra-broadband metamaterial absorbers from long to very long infrared regime
Source: Light Sci Appl. 2021 Jul 5;10:138. doi: 10.1038/s41377-021-00577-8 (PMC8257711; doi:10.1038/s41377-021-00577-8)
Supplement: Supplementary file 1 — Additional information for proposed absorbers [file 41377_2021_577_MOESM1_ESM.docx]

Supporting Information

Ultra-broadband metamaterial absorbers from long to very long infrared regime

*Yu Zhou^1,2^, Zheng Qin^1,2^, Zhongzhu Liang^1,2,3*^, Dejia Meng^1^, Haiyang Xu^3^, David R. Smith^4^ and Yichun Liu^3^*

**Correspondence: Zhongzhu Liang* (*liangzz@nenu.edu.cn*)

^1^*State Key Laboratory of Applied Optics, Changchun Institute of Optics, Fine Mechanics and Physics, Chinese Academy of Sciences, Changchun, Jilin, 130033, China*

^2^*University of the Chinese Academy of Sciences, China*

^3^*Center for Advanced Optoelectronic Functional Materials Research and Key Laboratory of UV Light-Emitting Materials and Technology of Ministry of Education, College of Physics, Northeast Normal University, Changchun 130024, China*

^4^*Center for Metamaterials and Integrated Plasmonics, Duke University, P.O. Box 90291, Durham, North Carolina 27708, USA.*

# Section 1. Comparison of representative theoretical and experimental works on the topic of MPAs.

**Table S1.** Comparison of representative works on the topic of MPAs.

| Work by | Device Configuration | Operation Bandwidth | Total Abs. | thickness | Materials Involved | Fabrication method |
| --- | --- | --- | --- | --- | --- | --- |
| *Ding et al.* (2011) [[1](#_Ding,_P._et)] | Embedded metal-dielectric structure | Two peaks  680 nm  760 nm | >99% | SiO_2_: 150 nm | Au  SiO_2_ | N/A |
| *Li* *et al.* (2014) [[2](#_Li,_W._et)] | MIM tri-layer | 400–800 nm | ≈90% | SiO_2_: 60 nm | TiN  SiO_2_ | EBL + e-beam deposition |
| *Massiot* *et al.* (2014) [[3](#_Massiot,_I._et)] | Embedded metal-dielectric structure | 450−830 nm | >80% | GaAs: 25 nm Si_3_N_4_: 80 nm | Ag  GaAs  Si_3_N_4_ | EBL + e-beam deposition |
| *Liu* *et al.* (2015) [[4](#_Liu,_Z._Q.)] | MIM tri-layer | 439–835 nm | >80% | SiO_2_: 40 nm | Au  SiO_2_ | ion beam sputtering |
| *Qian et al.* (2017) [[5](#_Qian_Q.,_Sun)] | Embedded metal-dielectric structure | 400–760 nm | >90% | Total: 200 nm | Cr  Si  SiO_2_ | double-beam interference lithography |
| *Lei* *et al.* (2018) [[6](#_Lei_L.,_Li)] | MIM tri-layer | 354–1066 nm | >90% | SiO_2_: 80 nm | Ti  SiO2  Al | N/A |
| *Wu* *et al.* (2019) [[7](#_Wu_S.,_Ye)] | Embedded metal-dielectric structure | 250–2250 nm | ≈87% | Si_3_N_4_: 160 nm | Si_3_N_4_  TiN | Photolithography  Etching  Deposition |
| *Mou et al.* (2020) [[8](#_Mou_N.,_Liu)] | MIM structure (Al/GST/Al) | 480–1020 nm | >80% | GST: 40 nm | Al_2_O_3_  Al  GST | Photolithography  Etching  Deposition |
| *Jiang* *et al.* (2020) [[9](#_Jiang_X.,_Wang)] | Laminated metal-dielectric structure | 300–2215 nm | >90% | SiO_2_: 70 nm TiO_2_: 15 nm | SiO_2_  TiO_2_  W | N/A |
| *Zhu et al.* (2020) [[10](#_Zhu_L.,_Jin)] | MIM tri-layer | 300–2000 nm | >90% | Al_2_O_3_: 100 nm | Ti  Al_2_O_3_ | N/A |
| *Yu et al.* (2020) [[11](#_Yu_P.,_Yang)] | MMIM four-layer | 166.8–1926.6 nm | >90% | SiO_2_: 260 nm | Ti, W  SiO_2_  Al | N/A |
| *Liu* *et al.* (2020) [[12](#_Liu_Y.,_Liu)] | Laminated metal-dielectric structure | 300–3000 nm | >96% | Total: 1485 nm | Fe  Si | N/A |
| *Yi* *et al.* (2020) [[13](#_Yi_Z.,_Li)] | Embedded metal-dielectric structure | 420–1950 nm | >90% | SiO_2_: 140 nm | W  SiO_2_ | N/A |
| *Liu et al.* (2010) [[14](#_Liu_X.,_Starr)] | MIM tri-layer | One peak  6 μm | >95% | SiO_2_: 300 nm | Au  SiO_2_ | EBL + e-beam deposition |
| *Hao et al.* (2010) [[15](#_Hao_J.,_Wang)] | MIM tri-layer | One peak  1.58 μm | ≈88% | Al_2_O_3_: 10 nm | Au  Al_2_O_3_ | EBL + e-beam deposition |
| *Feng et al.* (2014) [[16](#_Feng_R.,_Qiu)] | 2×2 Five-layer multi-sized structure | 8–12 μm | >90% | Ge: 500 nm  ZnTe: 300 nm | Ag  ZnTe  Ge | N/A |
| *Raman et al.* (2014) [[17](#_Raman,_A.,_Anoma,)] | Mutilayer structure | 8−12 µm | ≈70% | Total dielectric (HfO_2_+SiO_2_): 1577 nm | HfO_2_  SiO_2_  Ti  Ag | Deposition |
| *Guo et al.* (2016) [[18](#_Guo_W.,_Liu)] | 2×2 Five-layer multi-sized structure | 5.2−13.7 µm | >80% | Al_2_O_3_: 670 nm  ZnS: 590 nm | Al_2_O_3_  Ag  ZnS | N/A |
| *Hasan et al.* (2017) [[19](#_Hasan_D.,_Pitchappa)] | MIM tri-layer | 5.5−6.5 µm | >50% | AlN: 200 nm | Mo  AlN | EBL + e-beam deposition |
| *Zhai et al.* (2017) [[20](#_Zhai_Y.,_Ma)] | Glass-polymer hybrid structure | 2.5−25 µm | ≈70% | TPX: 50 μm | TPX  SiO_2_  Ag | Deposition+  gravimetric feeders |
| *Li et al.* (2018) [[21](#_Li_Z.,_Stan)] | 2×2 multi-sized MIM structure | 3.5−5.5 µm | >80% | Al_2_O_3_: 200 nm | W  Al_2_O_3_ | EBL + e-beam deposition |
| *Shrestha et al.* (2018) [[22](#_Shrestha_S.,_Wang)] | MIM structure (ITO/Si/ITO) | 4−16 µm | ≈80% | Dielectric: 1400 nm | ITO  Si  Oxide | EBL + e-beam deposition |
| *Lei* *et al.* (2019) [[23](#_Lei_L.,_Lou)] | MIM tri-layer | 1627– 4696 nm | >80% | VO_2_: 260 nm | Cr  VO_2_ | N/A |
| *Fann et al.* (2019) [[24](#_Fann_C.,_Zhang)] | MIM tri-layer | 4.8−7.5 μm  9.7−10.5 µm | >80% | SiO_2_: 1000 nm | Ti  SiO_2_  Al | Photolithography + e-beam evaporation |
| *Luo et al.* (2019) [[25](#_Luo_Y.,_Liang)] | Embedded metal-dielectric structure | 8–14 μm | >80% | Ge: 1120 nm | Ti  Ge | N/A |
| *Luo et al.* (2019) [[26](#_Luo_Y.,_Meng)] | 2×2 Five-layer multi-sized structure | 8–14 μm | >90% | Ge: 1620 nm | Ti  Ge | N/A |
| *Hou* *et al.* (2020) [[27](#_Hou_E.,_Meng)] | MIMIM five-layer | 3–5 μm  8–14 μm | ≈65%  ≈65% | SiO_2_: 80 nm  Ge: 220 nm | Au  Ge  SiO_2_ | N/A |
| Our work | MIM tri-layer | 2.93 μm  8–12 μm | 95%  90% | Ge: 500 nm | Ti  Ge | EBL + e-beam deposition |
|  | MDDM four-layer | 8–14 μm  6.5–13.5 μm | 95%  78% | Ge: 270 nm  Si_3_N_4_:330 nm | Ti  Ge  Si_3_N_4_ | EBL + e-beam deposition |
|  | MDDM four-layer | 14–30μm  8–30μm | 92%  87% | Si+SiO_2_:  1.63 μm  Si+SiO_2_:  1.8 μm | Ti  Si  SiO_2_ | N/A |

# Section 2. Additional information of the Ti/Ge/Ti absorber


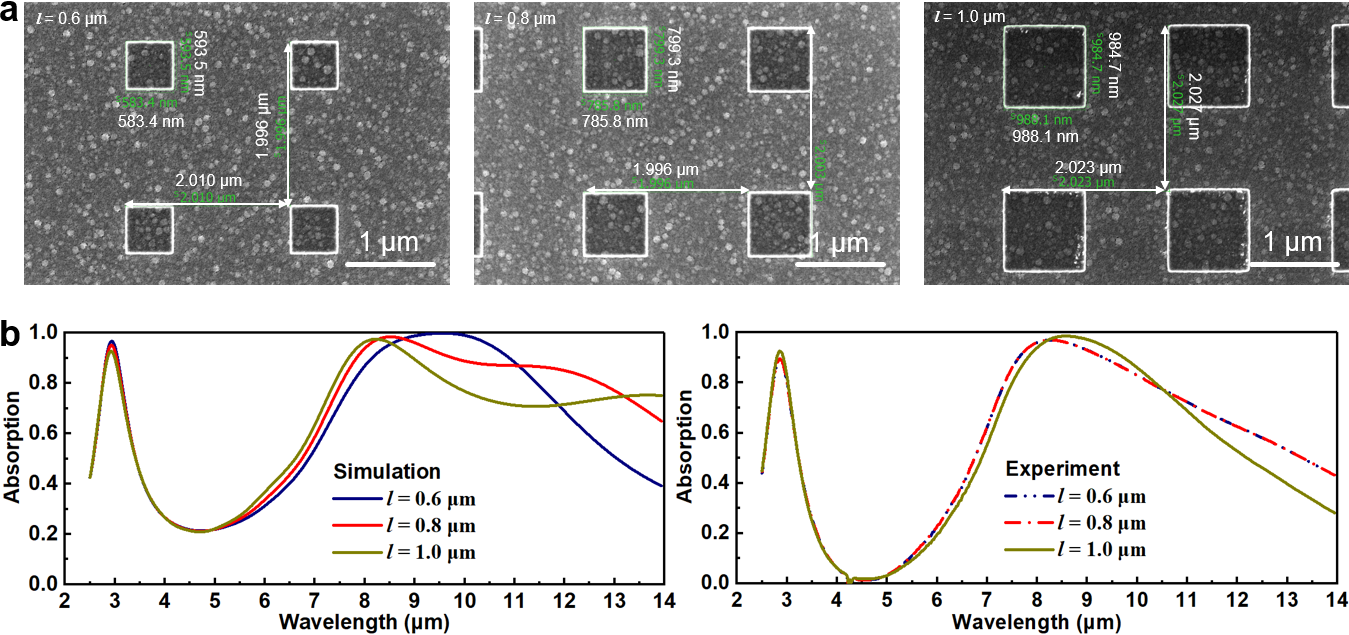


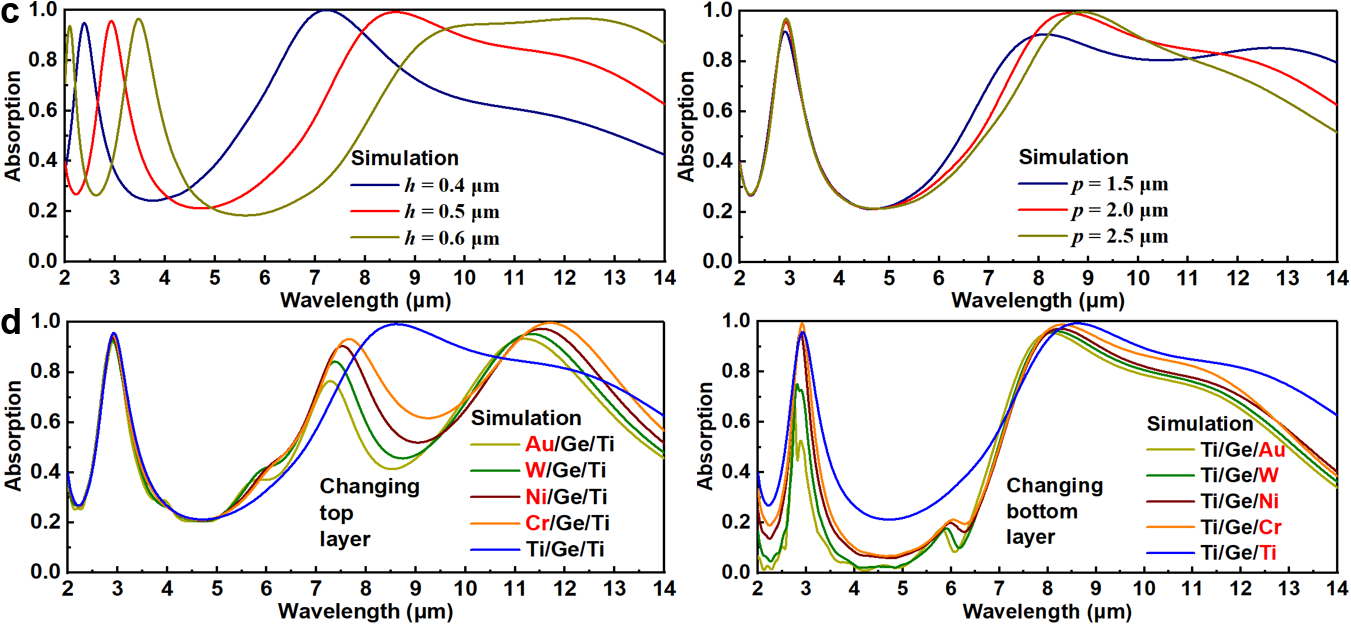


### **Fig. S1.** (a) SEM images of the fabricated Ti/Ge/Ti absorber with different lengths (0.6 μm, 0.8 μm and 1.0 μm) of the top nano-square resonators. (b) Simulated and experiment absorption spectra of the Ti/Ge/Ti absorber with different lengths (*l* = 0.6 μm, 0.8 μm and 1.0 μm) of the top nano-square resonators. (c) Simulated absorption spectra of the Ti/Ge/Ti absorber with different dielectric thicknesses (*h*) and periods (*p*). (d) Simulated absorption spectra of changing the top and the bottom layers with different metals.

Figure S1a shows the SEM images of the EBL fabricated Ti/Ge/Ti absorber with three different lengths (0.6 μm, 0.8 μm and 1.0 μm) of the top nano-square resonators which locate at the Ge layer, and Figure S1b shows the compared absorption spectra of the simulated and experiment results. The absorption dominated by the PSP and LSP resonances can be tuned flexibly by changing the various parameters of the structure. From the simulation results in Fig. S1b, it can be seen that adding the lengths (*l*) of the top nano-square resonators increases the LSP resonant wavelength. We find that the experiment results are not very sensitive to the parameters of the top Ti nano-square resonators. From Fig. S1c, the absorption peaks dominated by PSP and LSP resonances are sensitive to the thickness and period of the absorber. We can find that adding the dielectric thickness increases the total absorption band, which can be verified by interference theory^28^, adding the dielectric thickness increases a phase difference, then increasing the PSP and LSP resonant wavelengths. Moreover, changing the metallic layers can also affect the resonant wavelengths. According to Palik’s handbook^29^, refractory metals such as Ti and Cr have smaller refractive or permittivity values in infrared regime than traditional noble metals, such as Au and Ag. For our referred five metals in Fig. S1d, the refractive or permittivity order of value is Au>W>Ni>Cr>Ti. According to the theoretical analyses, it can be found that the absorption characteristics are based on the parameters of the metamaterial absorber. Due to the complex absorption mechanisms, we may hardly calculate the absorption peaks of the absorber accurately, but we can get some regularities such as choosing which materials and which parameters make the absorber achieve our requirements. Because the LSP resonant mode can be explained by the parallel *RLC* circuit, we can introduce a parameter (*Q*_LSP_) which represents the quality factor of the LSP mode. *Q*_LSP_ can be expressed as:

$Q=\frac{1}{R}\sqrt{\frac{C}{L_{m}+L_{e}}}$ (S1)

The greater the *Q* factor, the better the selectivity, the higher the absorptivity and the sharper the absorption curve. Small permittivity metals correspond to low-*Q* resonances. Low-*Q* metals such as Ti and Cr have good potentials and shows better performances in consisting as metallic layers of infrared broadband MPA.

Table S2 shows the parameters of the MIM absorbers as they affect the absorption characteristics based on the theoretical background (|*ε*_mt_| and |*ε*_mb_| are the permittivity value of the top and bottom metallic layers, respectively; *ε_d_* is the dielectric permittivity; *h* is the dielectric thickness; and *p* is the period of the MPA):

# Table S2. Absorption characteristics affected by parameters of MPAs.

|  | **\|*ε_mt_*\|↑** | **\|*ε_mb_*\|↑** | **\|*ε_d_*\|↑** | ***h*↑** | ***p*↑** |
| --- | --- | --- | --- | --- | --- |
| *λ*_PSP_ | none | blueshift← | redshift→ | redshift→ | redshift→ |
| *λ*_LSP_ | blueshift← | blueshift← | redshift→ | redshift→ | blueshift← |
| *Q*_LSP_ | increase↑ | increase↑ | increase↑ | decrease↓ | increase↑ |

For the resonant wavelength in PSP mode: at first, according to the exciting condition of the PSP mode, the top layer of the MPA functions as a metallic grating that produces a scattering wave. The permittivity of the top layer will not affect the resonant wavelength of the PSP mode. However, changing the metal of the top layer does indeed affect the PSPR-dominated absorption, because the reflectivity of the incident wave at the top layer also depends on the refractive index of the metal. And changing the bottom metallic layer for higher-permittivity metals decreases the resonant wavelength of the PSP mode. When the scattering wave transmits through the insulator layer and then arrives at the interface between the bottom and the insulator to match the PSP, a phase difference is produced. Therefore, adding to the permittivity or thickness of the insulator increases the PSPR wavelength. For the period of the MPA, which can be regarded as a grating constant, adding the period means increasing the scatter angle and then the scatter wavevector, which then increases the PSPR wavelength.

For the resonant wavelength in LSP mode: at first, any change to the top and bottom layers with higher-permittivity metals can both decrease the inductance (*L*_e_) induced by the drifting electrons and then decrease the LSPR wavelength. Adding to the permittivity or thickness of the insulator increases the value of the interference term, which then increases the LSPR wavelength. However, for special absorbing structures with a super-thin dielectric–maybe less than 100 nm for infrared band or 10 nm for the visible band^30,31^–the effect of the interference term can be neglected, then the increase in the equivalent capacitance caused by decreasing the thickness of the dielectric mainly affects the LSP resonant wavelength, leading to a large resonant wavelength corresponding to small dielectric thickness. Then, it is easy to prove that adding to the period decreases the LSPR wavelength.

The quality factor of the LSP resonant mode depends on the absorption properties of the LSPR-dominated absorption. At the LSP resonant wavelength, the absorptivity of a large *Q*-factor LSP mode is higher than that of a small *Q*-factor LSP mode. Furthermore, the absorption peak of a large *Q*-factor LSP mode is sharp. Changing the top and the bottom layers with higher-permittivity metals decreases the inductance, which then increases the *Q*-factor. Adding to the dielectric permittivity increases equivalent capacitance, which then increases the *Q*-factor. However, adding to the thickness of the dielectric increases Faraday inductance, *L*_m_, due to the mutual effects and decreases the equivalent capacitance, *C*, for the insulator between the two metallic layers, which then decreases the *Q*-factor. And it can be easily verified that adding to the period increases the *Q*-factor.

# Section 3. Additional information of the Ti/Ge/Si_3_N_4_/Ti absorbers


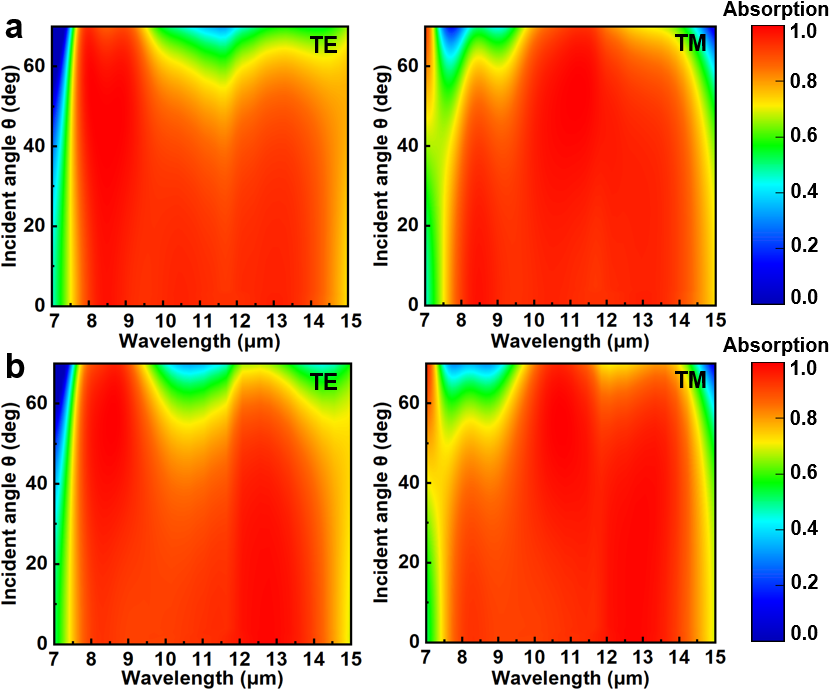


### **Fig. S2.** TE–mode (x–polarized) and TM–mode (y–polarized) absorption spectra for incident angles from 0–70°. (a) Ti/Ge/Si_3_N_4_/Ti absorber with periodic nano-cross and nano-strip top metallic resonators. (b) Ti/Ge/Si_3_N_4_/Ti absorber with periodic irregular nano-cross top metallic resonators.


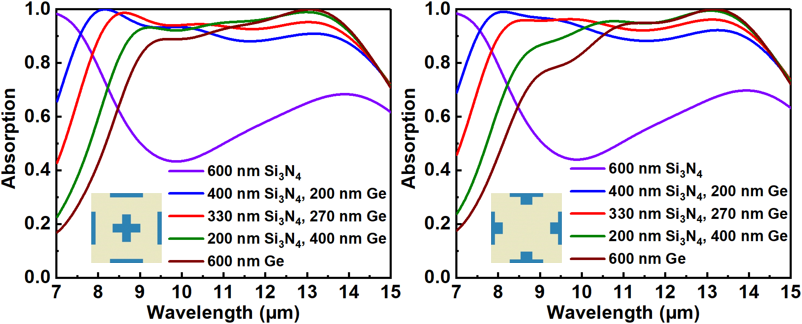


### **Fig. S3.** Absorption spectra of diﬀerent proportions of Si_3_N_4_- and Ge-layer thickness in the dielectric for the two Ti/Ge/Si_3_N_4_/Ti absorbers.


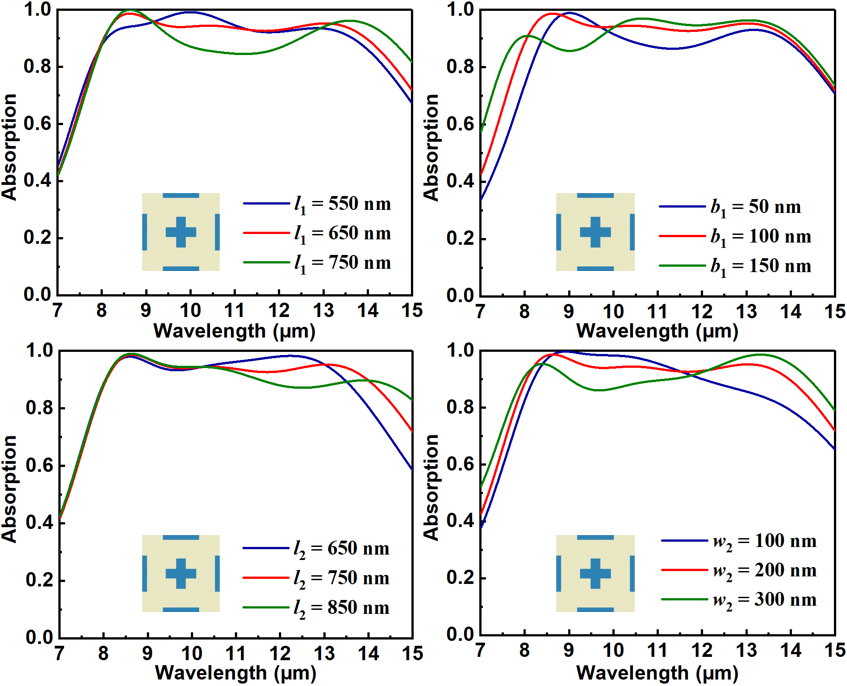


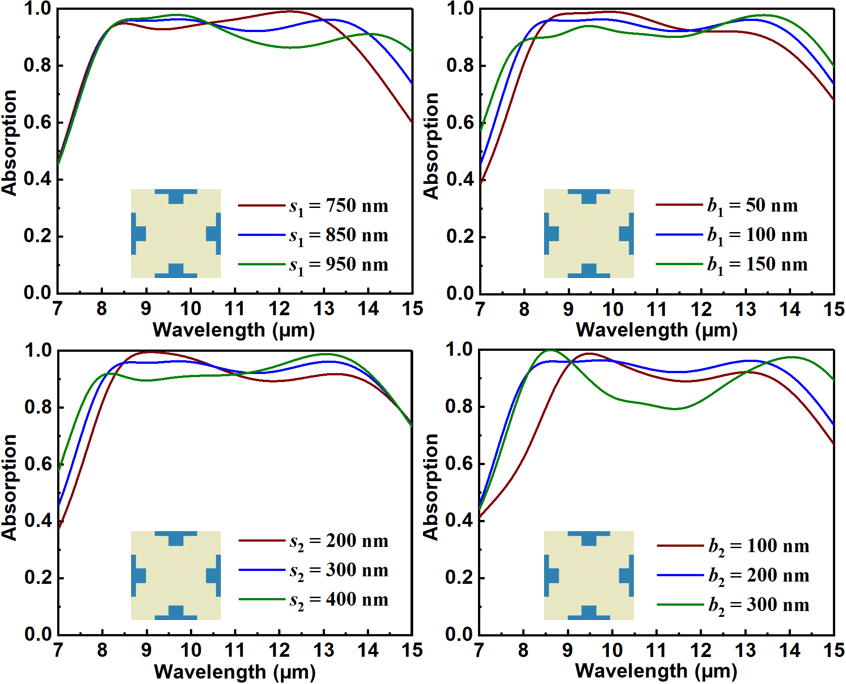


### **Fig. S4.** Absorption spectra of different parameters of the periodic top metallic structure of two Ti/Ge/Si_3_N_4_/Ti absorbers.

# Section 4. Additional information of the Ti/Si/SiO_2_/Ti absorbers

#### **Ti/Si/SiO_2_/Ti absorber from 14–30 μm**


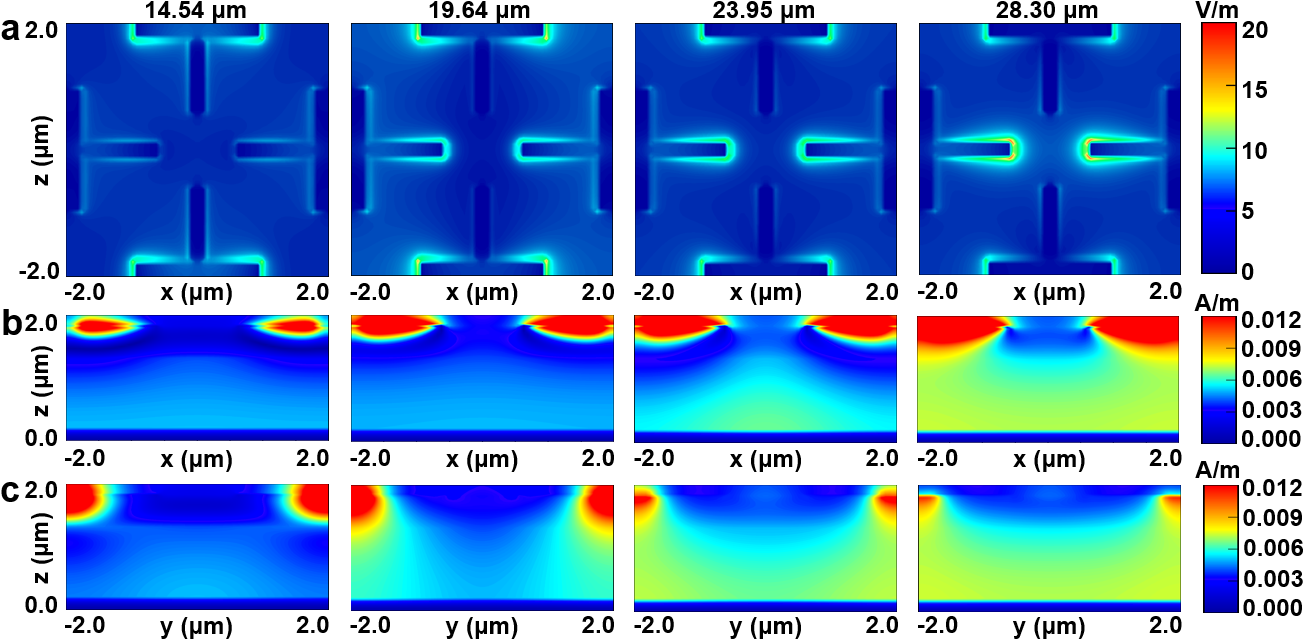


### **Fig. S5.** Electromagnetic field distributions of the Ti/Si/SiO_2_/Ti absorber at four resonant wavelengths along the VLWIR range: 14.54 μm, 19.64 μm, 23.95 μm and 28.30 μm. (a) Electric field distributions of top layer (x-y plane). (b) Magnetic field distributions in dielectric (x-z plane, y = 0). (c) Magnetic field distributions in dielectric (y-z plane, x = 0).


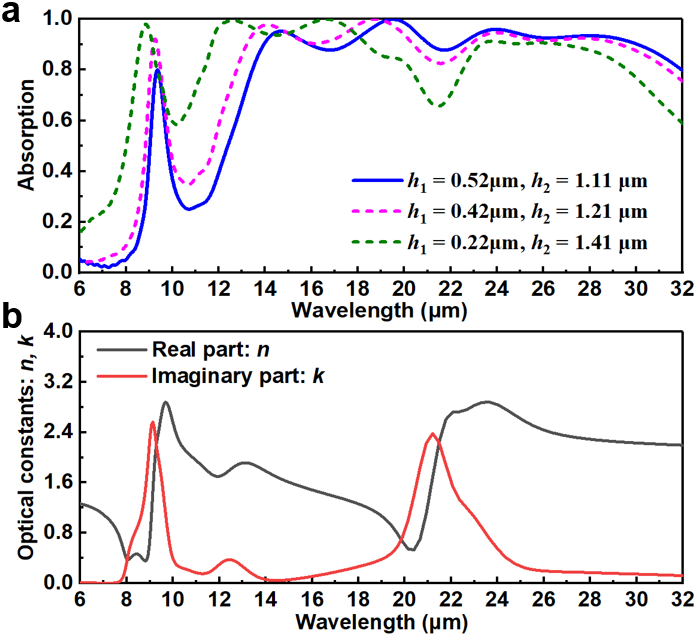


### **Fig. S6.** (a) Absorption spectra of diﬀerent thicknesses of Si and SiO_2_ in the dielectric. (b) Optical constants of SiO_2_ from long to very long infrared regime.

Figure S5a shows the magnetic field distributions within the top metallic layer at the four resonant wavelengths of 14.54 μm, 19.64 μm, 23.95 μm and 28.30 μm. As same as previous Ti/Ge/Si_3_N_4_/Ti absorber, it indicates that ultra-broadband absorption bandwidth is influenced by coupling hybrid resonances from the top cross resonators. Figure S5b and S5c shows the lateral magnetic field distributions in dielectric within x–z plane and y–z plane at the four resonant wavelengths, respectively. At the first absorption peak of 14.54 μm, which is dominated by two directions of PSP resonances. The resonances of two x-oriented cross resonators are weaker than two y-oriented cross resonators, then it can be seen that the PSPs resonance along the x direction (y-z plane) is excited more strongly than along the y direction (x-z plane). Then at the wavelength of 19.64 μm and 23.95 μm, PSPR and LSPR dominate y- and x-direction resonances, respectively. It means that the two absorption peaks of 19.64 μm and 23.95 μm are dominated by complementary resonances of y-direction PSPs and x-direction LSPs that can contribute commonly on absorption bandwidth. Then as the incident wavelength gradually increases, at the fourth peak of 28.30 μm, both x- and y-direction LSP resonances take over and dominate the absorptivity. The plasmon modes of two directions of the four absorption peaks are marked in Fig. S6a. It also indicates that such periodic irregular nano-cross top resonators can excite four resonant modes theoretically. Figure S6b shows optical constants of SiO_2_ from long to very long infrared regime, it can be seen that there are two peaks of imaginary-part refractive index in LWIR and VLWIR bands, respectively, which can excite intrinsic absorptions in target regimes.

#### **Ti/Si/SiO_2_/Ti absorber from 8–30 μm**


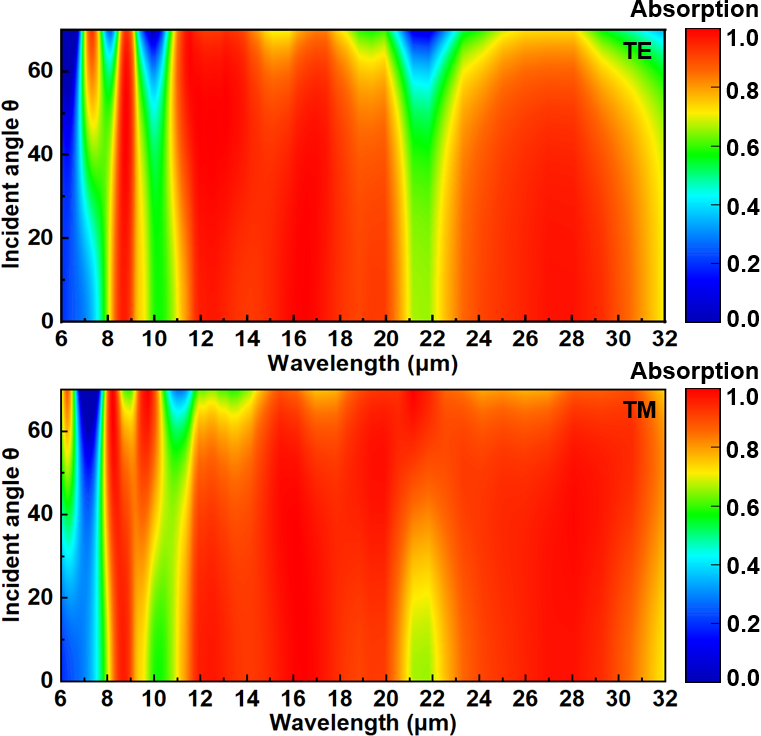


### **Fig. S7.** TE–mode (x–polarized) and TM–mode (y–polarized) absorption spectra of the 8–30 μm Ti/Si/SiO_2_/Ti absorber for incident angles from 0–70°.


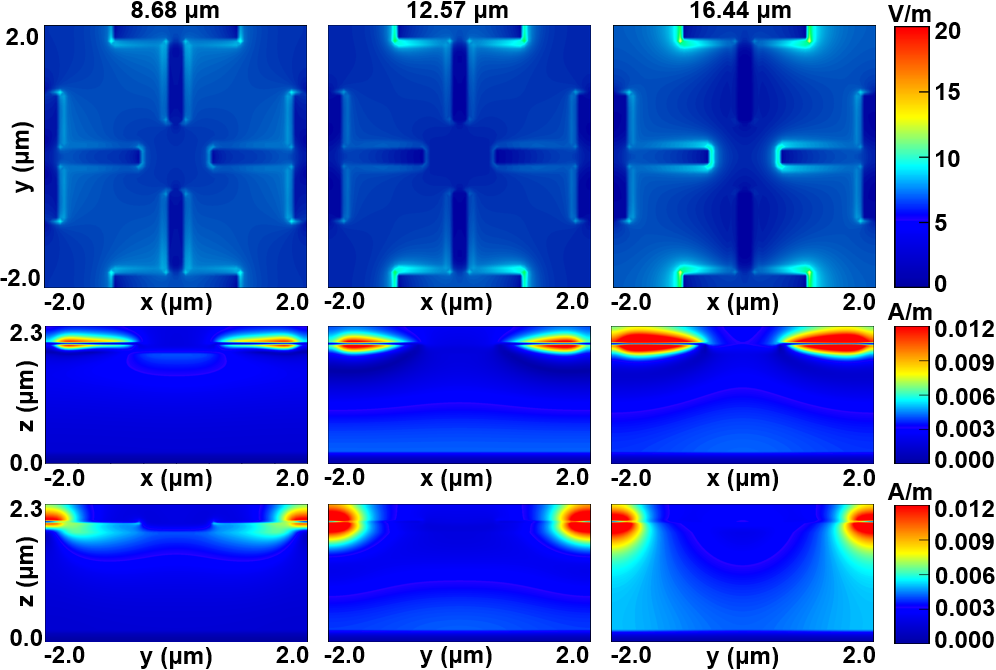


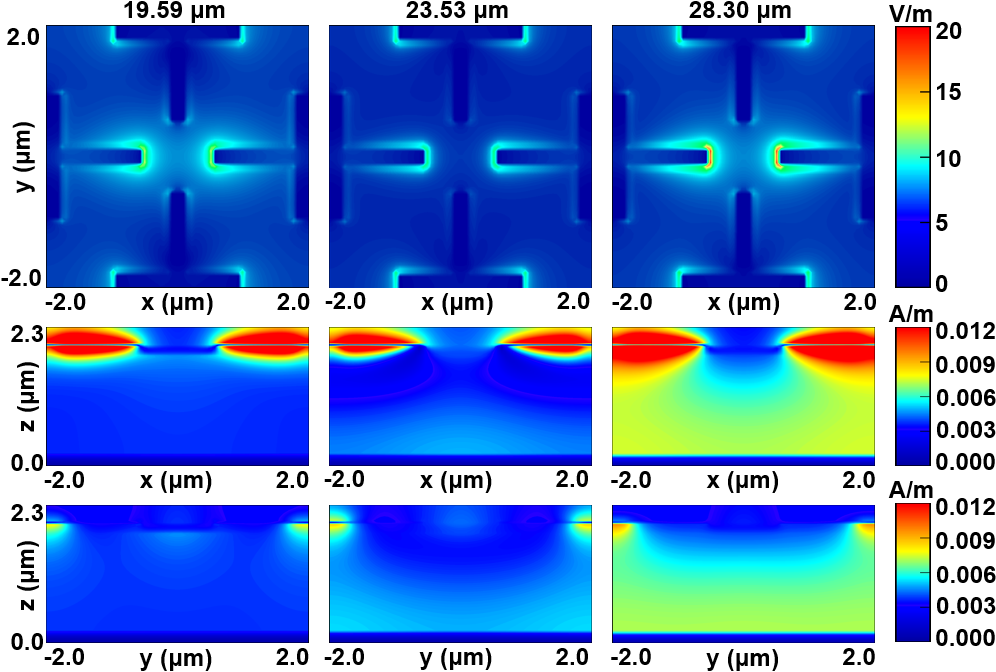


#### **Fig. S8.** Electromagnetic field distributions of the 8–30 μm Ti/Si/SiO_2_/Ti absorber at six resonant wavelengths along the LWIR and VLWIR range: 8.68 μm, 12.57 μm, 16.44 μm, 19.59 μm, 23.53 μm and 28.30 μm (Up: Electric field distributions of top layer (x-y plane); Middle: Magnetic field distributions in dielectric (x-z plane, y = 0); bottom: Magnetic field distributions in dielectric (y-z plane, x = 0))


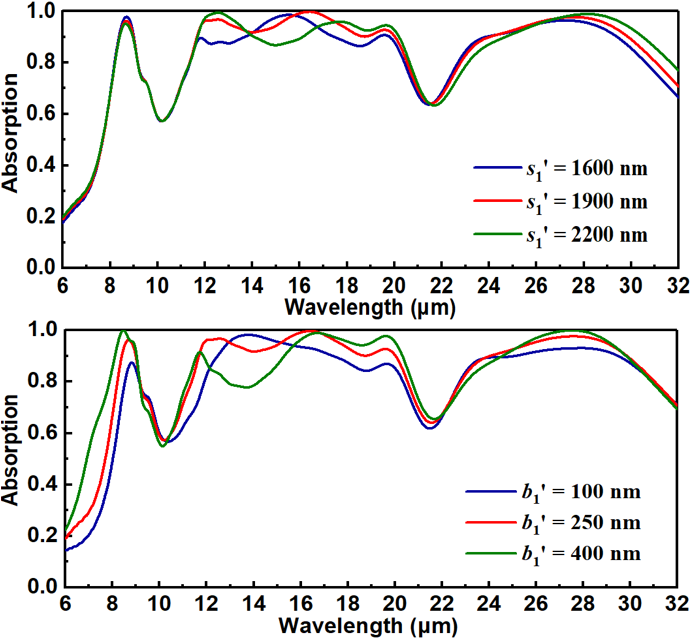


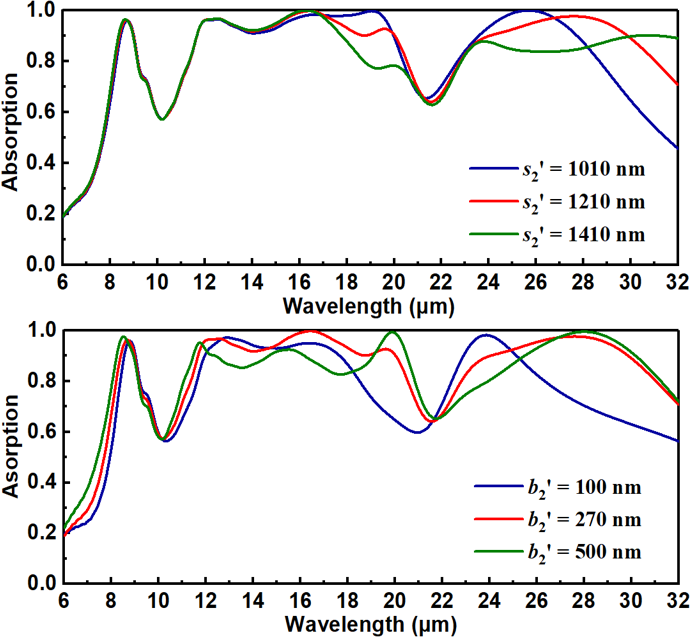


### **Fig. S9.** Absorption spectra of different parameters of the periodic top metallic structure of the 8–30 μm Ti/Si/SiO_2_/Ti absorber.

# References

#### Ding, P. *et al*. Dual-band perfect absorption and field enhancement by interaction between localized and propagating surface plasmons in optical metamaterials. *Journal of Optics* **13**, 075005 (2011).

#### Li, W. *et al*. Refractory plasmonics with titanium nitride: broadband metamaterial absorber. *Advanced Materials* **26**, 7959-7965 (2014).

#### Massiot, I. *et al*. Metal nanogrid for broadband multiresonant light-harvesting in ultrathin GaAs layers. *ACS Photonics* **1**, 878-884 (2014).

#### Liu, Z. Q. *et al*. Automatically acquired broadband plasmonic-metamaterial black absorber during the metallic film-formation. *ACS Applied Materials & Interfaces* **7**, 4962-4968 (2015).

#### Qian, Q. Y. *et al*. Large-area wide-incident-angle metasurface perfect absorber in total visible band based on coupled mie resonances. *Advanced Optical Materials* **5**, 1700064 (2017).

#### Lei, L. *et al*. Ultra-broadband absorber from visible to near-infrared using plasmonic metamaterial. *Optics Express* **26**, 5686-5693 (2018).

#### Wu, S. L. *et al*. Large-area, ultrathin metasurface exhibiting strong unpolarized ultrabroadband absorption. *Advanced Optical Materials* **7**, 1901162 (2019).

#### Mou, N. L. *et al*. Large-scale, low-cost, broadband and tunable perfect optical absorber based on phase-change material. *Nanoscale* **12**, 5374-5379 (2020).

#### Jiang, X. Y. *et al*. Ultrabroadband light absorption based on photonic topological transitions in hyperbolic metamaterials. *Optics Express* **28**, 705-714 (2020).

#### Zhu, L. *et al*. Ultra-broadband absorber based on metal-insulator-metal four-headed arrow nanostructure. *Plasmonics* **15**, 2153-2159 (2020).

#### Yu, P. Q. *et al*. Ultra-wideband solar absorber based on refractory titanium metal. *Renewable Energy* **158**, 227-235 (2020).

#### Liu, Y. Y. *et al*. Ultra-broadband perfect absorber utilizing a multi-size rectangular structure in the UV-MIR range. *Results in Physics* **18**, 103336 (2020).

#### Yi, Z. *et al*. Broadband polarization-insensitive and wide-angle solar energy absorber based on tungsten ring-disc array. *Nanoscale* **12**, 23077-23083 (2020).

#### Liu, X. L. *et al*. Infrared spatial and frequency selective metamaterial with near-unity absorbance. *Physical Review Letters* **104**, 207403 (2010).

#### Hao, J. M. *et al*. High performance optical absorber based on a plasmonic metamaterial. *Applied Physics Letters* **96**, 251104 (2010).

#### Feng, R. *et al*. Parallel *LC* circuit model for multi-band absorption and preliminary design of radiative cooling. *Optics Express* **22**, A1713-A1724 (2014).

#### Raman, A. P. *et al*. Passive radiative cooling below ambient air temperature under direct sunlight. *Nature* **515**, 540-544 (2014).

#### Guo, W. L., Liu, Y. X. & Han, T. C. Ultra-broadband infrared metasurface absorber. *Optics Express* **24**, 20586-20592 (2016).

#### Hasan, D. *et al*. Novel CMOS-compatible Mo-AlN-Mo Platform for metamaterial-based mid-IR absorber. *ACS Photonics* **4**, 302-315 (2017).

#### Zhai, Y. *et al*. Scalable-manufactured randomized glass-polymer hybrid metamaterial for daytime radiative cooling. *Science* **355**, 1062-1066 (2017).

#### Li, Z. G. *et al*. Wavelength-selective mid-infrared metamaterial absorbers with multiple tungsten cross resonators. *Optics Express* **26**, 5616-5631 (2018).

#### Shrestha, S. *et al*. Indium tin oxide broadband metasurface absorber. *ACS Photonics* **5**, 3526-3533 (2018).

#### Lei, L. *et al*. Tunable and scalable broadband metamaterial absorber involving VO_2_-based phase transition. *Photonics Research* **7**, 734-741 (2019).

#### Fann, C. H. *et al*. Broadband infrared plasmonic metamaterial absorber with multipronged absorption mechanisms. *Optics Express* **27**, 27917-27926 (2019).

#### Luo, Y. *et al*. Ultra-broadband and high absorbance metamaterial absorber in long wavelength Infrared based on hybridization of embedded cavity modes. *Optics Communications* **448**, 1-9 (2019).

#### Luo, Y. *et al*. Ultra-broadband metamaterial absorber in long wavelength Infrared band based on resonant cavity modes. *Optics Communications* **459**, 124948 (2020)

#### Hou, E. Z. *et al*. Mid-wave and long-wave infrared dual-band stacked metamaterial absorber for broadband with high refractive index sensitivity. *Applied Optics* **59**, 2695-2700 (2020).

#### Chen H. Interference theory of metamaterial perfect absorbers. *Opt. Express* **20**, 7165–7172 (2012).

#### Palik E. D. *Handbook of optical constants of solids,* (Academic, 1998).

#### Zhu J., Zhang L., Jiang S., Ou J., & Liu Q. H. Selective light trapping of plasmonic stack metamaterials by circuit design. *Nanoscale* **12**, 2057–2062 (2020).

#### Du K., Li Q., Zhang W., Yang Y., & Qiu M. Wavelength and Thermal Distribution Selectable Microbolometers Based on Metamaterial Absorbers. *IEEE Photon.* **7**(3), 6800908 (2015).
